# Variations in general practitioners’ referrals to low-value MRI examinations in Norway: A nationwide register-based study

**DOI:** 10.1080/13814788.2026.2706207

**Published:** 2026-08-10

**Authors:** Eivor H. Hoff, Bjørn M. Hofmann, Kristian B. Kraft, Arnstein Mykletun, Kristian A. Østby

**Affiliations:** ^a^ Norwegian Institute of Public Health (NIPH), Cluster for Health Services Research, Oslo, Norway; ^b^ Department of Community Medicine, UiT The Arctic University of Norway, Tromsø, Norway; ^c^ Office of the Auditor General of Norway (OAGN), Oslo, Norway; ^d^ Centre of Medical Ethics, The University of Oslo, Oslo, Norway; ^e^ Institute of the Health Sciences, The Norwegian University of Science and Technology (NTNU), Gjøvik, Norway; ^f^ Institute of Health and Society, University of Oslo, Oslo, Norway; ^g^ Nordland Hospital Trust, Centre for Work and Mental Health, Bodø, Norway; ^h^ Centre for Population Health, Haukeland University Hospital, Bergen, Norway

**Keywords:** Low-value care, MRI, referrals, general practitioners

## Abstract

**Background:**

Large variations in diagnostic imaging use raise concerns about low-value care, inequity, and inefficient use of resources. Magnetic resonance imaging (MRI) of the head, lumbosacral spine, and knee has been identified as potentially low-value examinations in several clinical contexts.

**Objectives:**

To examine variation in general practitioners’ referrals to low-value MRI examinations in Norway and assess whether differences are associated with GP and practice characteristics.

**Methods:**

Using national registry data, we linked patients’ completed MRI examinations to their regular GP and calculated GP-specific MRI rates per 1000 listed patients. We classified GPs into deciles and compared the characteristics of GPs in the highest (High-10) and lowest (Low-10) referral deciles. We examined predictors of variation using pooled OLS regression models with year fixed effects and GP-clustered standard errors.

**Results:**

GPs in the top referring decile (High-10) referred patients for potentially low-value MRI examinations at three times the rate of those in the lowest referring decile (Low-10). Experienced (specialist-certified) GPs referred approximately 2.7 fewer examinations per 1000 listed patients per year than less experienced GPs (non-specialists). Shorter consultation times, higher consultation frequency and higher hourly fee income were all associated with higher MRI use – with the strongest association for private MRI. A higher patient disease burden was associated with more total examination, but fewer private examinations per 1000 listed patients per year.

**Conclusion:**

Referral rates varied markedly between GPs. Specialist-certified GPs had lower rates than non-specialists, and higher activity levels and income from fees were associated with higher referral rates – particularly to private providers.

## KEY MESSAGES

- Substantial variation is found between GPs in referral rates to potential low-value MRI examinations.
- Specialist certification in general practice was a consistent predictor of lower referral rates.
- Higher activity levels and income from fees were positively associated with more referrals, particularly to private providers.

## Introduction

General practitioners (GPs) serve as the first point of contact for patients within many healthcare systems, performing initial assessments and treatments, and serving as gatekeepers to secondary care by deciding which patients require referrals to specialised examinations, such as advanced diagnostic imaging procedures.

Variation in GPs’ referral rates is expected due to differences in patient morbidity or access to health services. However, it may also reflect inconsistencies in clinical decision-making, with implications for quality of care, patient safety, and resource use. Systematic variation in referral rates across providers, after adjustment for patient need, indicates unwarranted variation in healthcare [1].

Unwarranted variations in referral rates undermine the ethical principle of justice [2] and challenge healthcare sustainability. These concerns are closely linked to low-value care, which broadly refers to care that is not clinically effective, not cost-effective, or potentially harmful. Based on the literature on low-value imaging [3–12], we define it as ‘imaging that does not contribute to reducing the overall pain, dysfunction or suffering of the person that is examined (compared to alternative actions)’ [13]. High patient demand and high GP referral volumes are drivers of low-value imaging [14–20], yet little is known about what drives variation in referral practices. A better understanding of GPs’ referral behaviour is therefore crucial.

In Norway, most GPs (∼ 85%) are self-employed and remunerated by a combination of fee-for-service (FFS) payments (per consultation and procedure, ∼70%) and capitation (CAP) payments (per enrolled patient, ∼30%). FFS/CAP payment model incentivises GPs to increase both their activity and patient volume. A minority of GPs (∼ 15%) are employed by the municipality and primarily receive a fixed salary irrespective of activity or patient volume.

All citizens have the right to be listed with a regular GP, and patients should normally be offered a GP consultation within five days [21]. There is a small co-payment for GP services, but primary care is predominantly publicly funded. All patients must obtain a referral to receive any MRI examination, and GPs are a major source of such referrals [22,23]. MRI examinations involve a patient co-payment, but patients may choose to pay the full cost out of pocket to access private providers directly, bypassing the public waiting list.

Patients may commonly request advanced imaging examinations, and GPs report that rejecting requests of doubtful usefulness is more time-consuming than accepting them [24], and around half of GPs report having sent unjustified referrals [25]. Time pressure and patient demand are among the most common reasons cited [24,25].

We focus on three specific imaging procedures identified as potentially low-value examinations, i.e. magnetic resonance imaging (MRI) of the head (caput), the lumbosacral spine (LS-spine) and the knee. More than half of knee MRI examinations [26] and 36% of the LS-spine in Norway [27] are most likely of low value. International literature has documented a high proportion of low-value MRI of the head [3] and of musculoskeletal MRI [28].

In this study, we address the following questions:

1. What is the variation in GPs’ referral rates to potentially low-value MRI examinations?
2. Which GP and practice characteristics are associated with variation in referral rates?

## Methods

### Data sources

We use rich, linked registry data covering the Norwegian population from the years 2016–2021. Information on patients’ MRI examinations is retrieved from the Norwegian Health Economics Administration’s (HELFO) KUHR database (Control and payment of health reimbursements) and includes all outpatient MRI examinations conducted at public hospitals or private providers. Only examinations eligible for public reimbursement are included.

Data on general practitioners (GPs) and their listed patients are retrieved from the Norwegian GP register (FLO) and supplemented by consultation-level data from the KUHR database. Sociodemographic variables are obtained from Statistics Norway (SSB). Patient diagnosis information was obtained from the Norwegian Patient Registry (NPR).

### Study population

We include GPs who are responsible for a patient list of at least 500 patients and who are actively practising (defined as conducting at least 15% of their listed patients’ contacts in a given month) for at least 10 out of 12 months. GPs are excluded in a given year if they have fewer than 100 working days that year, where a working day is defined as a weekday with at least five physical consultations. GPs are also excluded if they change remuneration scheme during the year. A total of 5347 unique GPs are included over the study period (∼70% of all GPs registered as regular GPs). We include all patients listed with each GP on 1 January each observation year. Listed patients refers to *residents* formally enrolled with a GP under Norway’s Regular GP scheme, which covers nearly the entire population, regardless of their healthcare use or morbidity.

### Statistical analyses

#### Outcomes

Our primary outcomes are GP-level referral rates to potentially low-value MRI examinations, calculated separately for all MRI (total rate) and MRI performed by private providers (private rate). We included MRI of the head, knee, and lumbosacral spine (Norwegian Classification of Radiological Procedures (NCRP) codes are SAA0AD (MRI Caput), SNG0AG (MRI Knee), and SNA0GG (MRI Lumbosacral-(LS)-spine)), as these examinations are identified as low-value examinations in several clinical contexts [2,29]. Not all such examinations are of low value; many are clinically warranted. We therefore refer to them as *potentially* low-value imaging. For each patient, we record whether they received at least one of these examination types during a given year. The patient’s regular GP is defined as the GP they were listed with per January 1st each observation year. All patient records of these examinations are then attributed to their regular GP. For descriptive analyses, age- and sex-standardised rates per 1000 listed patients are presented, using the 2016 Norwegian population as reference. In regression analyses, crude rates are used as outcomes, with mean patient age and share of female patients included as covariates. We distinguish between total and private MRI rates, as private providers may operate with different referral thresholds, making private MRI potentially more sensitive to GP-level variation in referral propensity and financial incentives.

#### Covariates

We distinguish between GP characteristics, practice characteristics, and patient list characteristics. All covariates are measured at the GP-year level.

As GP characteristics, we include GP sex, age in years, and specialist status. *Specialist status* is a binary variable indicating whether the GP holds a postgraduate specialisation in general practice, requiring a minimum of four years of supervised training in general practice.

As practice characteristics, we include remuneration scheme, consultation frequency, consultation duration, hourly income from fees, and patient list size. *Remuneration scheme* indicates whether the GP is salaried or remunerated through a combination of fee-for-service and capitation. *Consultation frequency* is the GP’s number of physical consultations (2AD) per listed patient for each GP. *Consultation duration* is an estimate of the average time spent on physical consultations with patients (see Supplementary material). In regression analysis, the variable is included as a binary variable (<20 min average consultation time). *Hourly income from fees* is the GP’s average hourly income from consultation and procedure fees during work hours (see Supplementary material). *Patient list size* represents the number of residents enrolled on the GP’s list (monthly average over the observation year).

As patient list characteristics, we include mean age of patients, share of female patients, share of patients with higher education (completed three years or more of university or college education), practice centrality, and mean comorbidity patients. *Practice centrality* is the mean centrality index of patients’ municipalities (Statistics Norway), with higher values indicating more urban areas. *Comorbidity* is measured using the Charlson Comorbidity Index (CCI), using Quan ICD-10 weights with a three-year roll-back period, i.e. based on ICD-10 diagnosis codes observed in the three years prior to each observation year.

#### Descriptive analyses

Descriptive statistics for the study population are presented for 2021. GPs are stratified into the lowest 10 percent (bottom decile, ‘Low-10’), the middle 80 percent (deciles 2 through 9, ‘Mid-80’) and the top 10 percent (top decile, ‘High-10’) of referrers to illustrate how characteristics differ across the referral rate distribution.

#### Regression models

We estimate associations between nine GP-level predictors and the two outcomes, the total MRI rate and the private MRI rate. The predictors were grouped as GP characteristics (specialist status, sex and age), practice characteristics (remuneration scheme, fee income per hour, short consultation time (average <20 min), consultation frequency, and patient list size), and patient list morbidity (mean Charlson Comorbidity Index).

Each predictor was examined in a separate set of three models, estimated separately for the two outcomes. Model 1 estimates the crude association between the main predictor and the outcome, Model 2 adjusts for GP characteristics and remuneration scheme (salaried vs. fee-for-service/capitation), and Model 3 additionally adjusts for patient list characteristics. When a covariate was itself the main predictor in a given model set, it was excluded from the adjustment set. Hourly income from fees was analysed among fee-for-service/capitation GPs only. The stepwise structure allows us to assess how much of each crude association is accounted for by GP and patient list characteristics.

All models were estimated using pooled ordinary least squares (OLS) regression with year fixed effects and standard errors clustered at the GP level. Outcomes were log-transformed to account for right-skewed distributions. OLS provides directly interpretable estimates of associations and performs well in large samples without strong distributional assumptions [40]. Year fixed effects account for common time trends, and clustering accounts for serial correlation due to repeated measures within GPs [41]. All continuous variables were standardised to SD = 1 to facilitate comparison of associations across predictors. Coefficients can be interpreted as approximate percentage differences in referral rates, per SD increase for continuous predictors and relative to the reference category for binary predictors. Estimates for main predictors are presented in Table 2. Estimates for all included covariates are presented in Supplementary material.

### Ethics

Data were collected in accordance with ethics approval from the regional ethics committee (REC, number 210548). The Norwegian Institute of Public Health conducted a privacy impact assessment regarding the data used.

## Results

Over the study period (2016–2021), 5347 unique GPs contributed 21 970 GP-years of observations. In 2021, the mean age of GPs was 49.4 years (SD 10.7), 57% were male, 71% held specialist certification in general practice, and 9% were salaried. In total, 1 276 403 MRI examinations (of which 74% were performed by private providers) were recorded for 1 076 944 unique patients.

**Figure 1.**
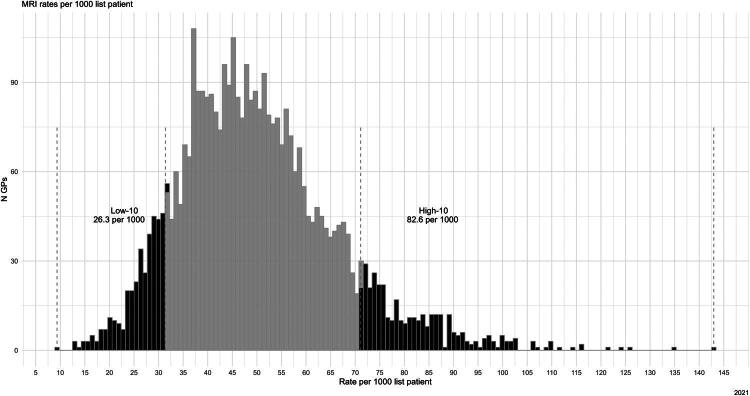
Histogram of MRI rates per 1000 list patients for GPs.

Figure 1 shows the distribution of GPs’ age- and sex-standardised MRI rates per 1000 listed patients in 2021. GPs in the highest decile (High-10) referred an average 82.6 examinations per 1000 listed patients, compared with 26.3 in the lowest decile (Low-10); a threefold difference.

Table 1 presents GP and patient list characteristics for the full 2021 population and by referral groups. Compared with Low-10 GPs, High-10 GPs were less often specialists (64% vs. 75%), less often salaried (3% vs. 15%), and more often male (61% vs. 54%). They conducted more consultations per patient (2.23 vs. 2.00), had shorter consultation times (18.6 vs. 19.9 min), larger lists (1180 vs. 1049 patients), and higher hourly income from fees (1179 vs. 1073 NOK). High-10 GPs also referred a higher share of examinations to private providers (74% vs. 68%). Patient list characteristics differed modestly: High-10 GPs practiced in more urban areas (centrality index 847 vs. 776), had slightly younger patients (40 vs. 42 years), a lower share of highly educated patients (34% vs. 37%), and a lower share of patients in the top income quintile (17% vs. 19%). Comorbidity burden and sex composition were virtually identical across groups.

**Table 1.** GP and patient list characteristics by MRI referral rate group, 2021. Groups are GPs in the lowest referral decile (Low-10), deciles 2–9 (Mid-80), and the highest referral decile (High-10).

	All	Low-10	Mid-80	High-10
	N = 3697	N = 370	N = 2957	N = 370
*Referral rates*				
Standardised MRI rate per 1000 listed patients (SD)	50.0 (16.1)	26.3 (4.2)	49.0 (10.0)	82.6 (11.1)
Share of private examinations (SD)	69 (25)	68 (27)	68 (25)	74 (23)
*GP characteristics*				
Specialist in general practice (%)	71	75	71	64
Male (%)	57	54	56	61
Age in years (SD)	49.4 (10.7)	48.7 (10.6)	49.5 (10.7)	48.6 (11.0)
*Practice characteristics*				
Salaried (%)	9	15	9	3
Hourly income from fees (SD)*	1 119 (252)	1 073 (247)	1 117 (249)	1 178 (268)
Average consultation duration (SD)	19.2 (4.2)	19.9 (4.4)	19.2 (4.2)	18.6 (4.1)
Consultation frequency (SD)	2.02 (0.65)	2.00 (0.63)	2.00 (0.64)	2.22 (0.75)
Patient list size (SD)	1 140 (322)	1 049 (316)	1 147 (322)	1 180 (314)
*Patient list characteristics*				
Mean practice centrality (SD)	817 (123)	776 (127)	818 (120)	847 (117)
Mean age in years (SD)	41 (6)	42 (6)	41 (5)	40 (6)
Share of female patients (SD)	50 (10)	50 (11)	50 (10)	50 (9)
Share with higher education (SD)	37 (14)	37 (14)	37 (14)	34 (13)
Share in highest income quintile (SD)	20 (8)	19 (7)	20 (8)	17 (7)
Mean Charlson Comorbidity Index (SD)	0.15 (0.05)	0.15 (0.05)	0.15 (0.05)	0.14 (0.05)

Note: Mean (SD) are presented for continuous variables, percentages for binary variables (specialist, male, salaried). Variables expressed as shares (private examinations, female patients, higher education, highest income quintile) are continuous at the GP level and presented as mean (SD) across GPs. Patient list characteristics are GP-level means of patient-level values, averaged across GPs within each group. *Salaried GPs are excluded and specialist-specific fees are subtracted from income calculations. Income values are in NOK (Norwegian krone).

Table 2 presents associations between each main predictor and the total and private MRI referral rates across three model specifications: crude (Model 1), adjusted for GP characteristics (age, sex, specialist status) and remuneration scheme (Model 2), and additionally adjusted for patient list characteristics (Model 3).

**Table 2.** Associations between GP characteristics, practice characteristics, and patient list morbidity and potentially low-value MRI referral rates, 2016–2021: crude (Model 1) and adjusted (Models 2 and 3) estimates.

	Total MRI rate			Private MRI rate		
	Model 1	Model 2	Model 3	Model 1	Model 2	Model 3
Main predictor	Crude	+ adjustments for GP characteristics	+ adjustments for patient list characteristics	Crude	+ adjustments for GP characteristics	+ adjustments for patient list characteristics
*GP characteristics*						
Specialist in general practice	−0.049*** (0.009)	−0.077*** (0.010)	−0.056*** (0.009)	0.023 (0.032)	−0.076* (0.035)	−0.114*** (0.032)
Male	0.050*** (0.009)	0.044*** (0.009)	0.048*** (0.011)	0.063† (0.032)	0.029 (0.032)	0.109* (0.044)
Age (per SD)	0.012** (0.004)	0.022*** (0.005)	0.002 (0.005)	0.063*** (0.015)	0.059*** (0.017)	0.008 (0.016)
*Practice characteristics*						
Salaried GP	−0.101*** (0.014)	−0.104*** (0.014)	−0.050*** (0.015)	−0.749*** (0.079)	−0.740*** (0.079)	−0.095 (0.073)
Short consultations (average < 20 min.)	0.089*** (0.009)	0.081*** (0.009)	0.040*** (0.009)	0.368*** (0.034)	0.287*** (0.035)	0.123*** (0.031)
Consultation frequency (per SD)	0.078*** (0.004)	0.073*** (0.004)	0.048*** (0.004)	0.106*** (0.014)	0.078*** (0.014)	0.072*** (0.014)
Hourly income from fees (per SD)*	0.050*** (0.005)	0.047*** (0.005)	0.030*** (0.004)	0.158*** (0.014)	0.158*** (0.014)	0.066*** (0.013)
Patient list size (per SD)	0.030*** (0.005)	0.024*** (0.005)	0.032*** (0.005)	0.266*** (0.015)	0.240*** (0.016)	0.050*** (0.014)
*Patient list morbidity*						
Mean Charlson Comorbidity Index (per SD)	0.065*** (0.004)	0.063*** (0.005)	0.037*** (0.007)	−0.124*** (0.017)	−0.136*** (0.018)	−0.127*** (0.025)

Note: β (SE) shown. † *p* < 0.1, * *p* < 0.05, ** *p* < 0.01, *** *p* < 0.001. Outcomes are log-transformed crude referral rates to potentially low-value MRI examinations per 1000 listed patients. Estimates (exp(β) − 1) × 100 gives the approximate percentage difference in the referral rate per SD increase for continuous predictors (standardised to SD = 1) or relative to the reference category for binary predictors. All models are pooled OLS with year fixed effects and standard errors clustered at the GP level. Each main predictor is examined in separate models. Model 1: crude association. Model 2: adjusted for GP sex, age, specialist status, and remuneration scheme. Model 3: additionally adjusted for mean patient age, share of female patients, share of patients with higher education, practice centrality, and mean Charlson Comorbidity Index. When a covariate is the main predictor in a given row, it is excluded from the adjustment set for that row. *N* = 21 764 GP-years. Overall R² in the fully adjusted models ranges from 0.129 to 0.144 (total MRI rate) and from 0.168 to 0.179 (private MRI rate). Full estimates for all covariates and model-specific R² are presented in the Supplementary material (Tables S1–S9). *Estimated for fee-for-service/capitation GPs only (*N* = 20 241 GP-years), the remuneration scheme (salaried vs. fee-for-service/capitation) covariate is not included in these models.

*GP specialists* had a lower total MRI rate than non-specialists in both crude and adjusted models: 5.4% lower total MRI rate in the fully adjusted model (β = −0.056***). For private MRI rate, the crude association was negligible (β = 0.023), however a negative association emerged after adjusting for GP characteristics (β = −0.076*) and further strengthened after adjustment for patient list (β = −0.114***), i.e. 10.7% lower private MRI rate.

*Male GPs* had a higher total MRI rate than female GPs in both crude and adjusted models: 4.9% higher total MRI rate in the fully adjusted model (β = 0.048***), and an 11.5% higher private MRI rate (β = 0.109*). For *GP age*, initial positive and significant associations in Models 1 and 2 were explained by patient list characteristics in Model 3, resulting in no significant associations.

*Salaried GPs* had a 10% lower crude MRI rate compared to fee-for-service/capitation GPs (β = −0.101***), reduced to about half after patient list adjustment (β = −0.050***). For private MRI, the crude association was stronger (β = −0.749***), however largely explained by patient list characteristics (β = −0.095, not significant).

All GP practice characteristics were independently associated with MRI rates after full adjustment. GPs with shorter consultation times had 4.1% higher total and 13.1% higher private MRI rates than those above or on the 20 min threshold (Model 3 β = 0.040*** and 0.123***). A one SD increase in consultation frequency was associated with 4.9% higher total and 7.5% higher private rates. For *hourly income from fees* (only fee-for-service GPs), a 1 SD increase in hourly income from fees was associated with a 3% higher total rate (β = 0.030***) and 6.8% higher private rate (β = 0.066***) in the fully adjusted Model 3.

For patient list morbidity, a 1 SD increase in mean Charlson Comorbidity Index was associated with a 3.8% *higher* total MRI rate and a 12% *lower* private MRI rate (Model 3: β = 0.037*** and −0.127***), the latter being the most robust across all model specifications.

The models explained a moderate share of between-GP variation in MRI referral rates, with overall R^2^ ranging from 13 to 14% for the total rate and 18% for the private rate.

## Discussion

This study documents substantial variation between GPs in referral rates to potentially low-value MRI. GPs in the highest referral decile (High-10) referred patients at approximately three times the rate of those in the lowest decile (Low-10). In 2021, around 1 in 12 patients listed with High-10 referrers underwent a potentially low-value MRI examination, compared with 1 in 40 among Low-10 referrers. Our results complement previous studies’ findings on variations in radiological examinations in Norway [30,31] and internationally [3], and on referral rates and quality [32].

We examined associations between a range of GP and GP practice characteristics and potentially low-value MRI referral rates. *GP specialists* had a 5.4% (total) and 10.7% (private) lower rate than non-specialists, corresponding to approximately 2.7 and 3.8 fewer examinations per 1000 listed patients per year. Specialist certification requires four years of postgraduate training and reflects both expertise and clinical seniority. This may reflect improved clinical decision-making, higher tolerance for diagnostic uncertainty, and greater familiarity with guidelines [23]. Male GPs referred more than female GPs to both total and private MRI, with the strongest association for private MRI.

Salaried GPs had a 10% lower total MRI rate than fee-for-service/capitation GPs, while a large crude association with private rate was fully explained by patient list characteristics. GPs with average consultation times below 20 min referred approximately 2.1 (total) and 4.6 (private) more examinations per 1000 listed patients per year for total MRI, compared to those with longer consultations. All other GP practice characteristics were also independently positively associated with MRI use. These findings may reflect that high consultation volumes and shorter visits reduce time for clinical assessments and discussions of uncertainty [19,24,33]. Financial incentives in a fee-for-service system may amplify such effects [19,24]. Our findings complement existing evidence that practice organisation is associated with low-value imaging [34], consistent with variation in these examinations partly reflecting supply-sensitive care, where provider preferences and practice styles play a central role [35].

For patient list morbidity, we found opposite associations for total and private MRI rate. A one SD increase in mean Charlson Comorbidity Index was associated with 1.9 more examinations per 1000 listed patients per year for total MRI, but 4.2 *fewer* private MRI. This suggests that sicker patients are mainly channelled to public specialist care, while healthier patients with weaker indications are directed to private MRI, possibly reflecting less stringent intake criteria among private providers.

Large variation in MRI referrals may reflect arbitrariness, inequity, and unfairness [2], and may threaten the quality of care. The effectiveness of interventions to reduce low-value referral varies [36], and guidelines alone have not consistently changed practice [37–39], suggesting that structural and organisational factors may be important targets for intervention. Our study explained 13--14% of between-GP variation in total MRI rates and 18% in the private MRI rate. A considerable proportion of between-GP variation remains unexplained, likely reflecting unmeasured factors such as tolerance for diagnostic uncertainty, patient preferences, and availability of alternative diagnostic pathways.

### Strengths and limitations

A strength of this study is the use of comprehensive national register data linking individual-level patient data to their regular GPs.

A limitation is that we observe completed MRI examinations rather than referrals, attributed to the patient’s regular GP, which may over- or underestimate a GP’s true referral rate. We argue that this approximation is unlikely to generate substantial systematic bias in comparisons between GPs. Additionally, self-funded private MRI examinations are not captured, likely underestimating private MRI use, particularly among wealthier and more highly educated patients.

Another limitation is the classification of the outcome. Classifying MRI of the head, knee, and lumbosacral spine rests on evidence that some of these examinations is of low value [3,26–28] and does not imply that every such examination is inappropriate. We cannot assess the appropriateness of individual examinations, and some of the variation observed will reflect legitimate differences in clinical need. Our estimates should therefore be interpreted not as direct measures of inappropriate care, but rather as variation in referral rates to types of examination with a high probability of low value.

Finally, regression analyses are observational and describe associations rather than causal effects. Despite adjusting for a range of GP, practice, and patient list characteristics, we cannot rule out that unmeasured confounding may affect the estimates.

## Conclusion

Referral rates to potentially low-value MRI examinations vary markedly between GPs. GP specialists had lower rates than non-specialists, and higher activity levels and income from fees were positively associated with higher referrals rates -- particularly to private providers.

## Supplementary Material

Supplemental Material

## Data Availability

The data that support the findings of this study are available from *Helsedataservice* and *Statistics Norway.* Restrictions apply to the availability of these data, which were used under licence for this study.
