# Supplementary material for: Variations in general practitioners’ referrals to low-value MRI examinations in Norway: A nationwide register-based study
Source: Eur J Gen Pract. 2026 Aug 10;32(1):2706207. doi: 10.1080/13814788.2026.2706207 (PMC13459299; doi:10.1080/13814788.2026.2706207)
Supplement: Supplementary file 1 [file IGEN_A_2706207_SM2397.pdf]

Supplementary Material

Variation in general practitioners' referrals to low-value MRI examinations in Norway: a nationwide register-based study

Supplementary Methods

Estimation of consultation duration and hourly income from fees.

Estimation of consultation duration

Consultation duration is measured as the time difference between the recorded start time of a physical patient consultation and the start time of the subsequent physical consultation. The estimation is restricted to daytime consultations occurring between 09:00 and 14:59 on workdays (weekdays with at least five physical consultations), and to consultations that are directly followed by either another physical consultation or a telephone consultation. At the GP level, days on which the GP conducted home visits before 15:00, and days on which 80 % or more of the GP's consultations began at standardised intervals (e.g. 09:00, 09:10, 09:20), are excluded. Average consultation duration is then estimated as the mean of all consultations meeting these inclusion criteria, per GP and year.

Estimation of hourly income from fees

Hourly income from fees is the average hourly income from consultation and procedure fees registered between 09:00 and 13:59 on workdays on which the GP worked three hours or more. Specialist-specific tariffs are excluded when calculating income from fees, to ensure comparability between GPs with and without specialist certification in general practice. Income from fees is not estimated for salaried GPs.

Supplementary Tables

Tables S1–S9: Full regression estimates (Models 1–3) for each main predictor – GP specialist status (S1), GP sex (S2), GP age (S3), remuneration scheme (S4), hourly income from fees (S5), consultation duration (S6), consultation frequency (S7), patient list size (S8), and patient list morbidity (S9).

Table S1. Associations between GP specialist status and potential low-value MRI referral rates, Models 1–3.

Variable	Total MRI rate			Private MRI rate		
	**Model 1**	**Model 2**	**Model 3**	**Model 1**	**Model 2**	**Model 3**
GP specialist (non-specialist)	-0.049*** (0.009)	-0.077*** (0.010)	-0.056*** (0.009)	0.023 (0.032)	-0.076* (0.035)	-0.114*** (0.032)
GP male (female)		0.044*** (0.009)	0.048*** (0.011)		0.029 (0.032)	0.109* (0.044)
GP age (per SD)		0.022*** (0.005)	0.002 (0.005)		0.059*** (0.017)	0.008 (0.016)
GP salaried (fee-for-service/capitation)		-0.104*** (0.014)	-0.050*** (0.015)		-0.740*** (0.079)	-0.095 (0.073)
Mean patient age (per SD)			0.019* (0.008)			0.088*** (0.027)
Share of female patients (per SD)			0.048*** (0.006)			0.079*** (0.020)
Share of patients with higher education (per SD)			-0.112*** (0.005)			-0.277*** (0.013)
Mean Charlson Comorbidity Index (per SD)			0.037*** (0.007)			-0.127*** (0.025)
Practice centrality (per SD)			0.097*** (0.006)			0.587*** (0.023)
R^2 (overall)	0.011	0.026	0.129	0.003	0.030	0.177
N (GP-years)	21 764	21 764	21 764	21 764	21 764	21 764

Note: β (standard error) shown. † p<0.1, * p<0.05, ** p<0.01, *** p<0.001. Outcomes are log-transformed crude referral rates to potentially low-value MRI examinations per 1000 listed patients. All models are pooled OLS with year fixed effects and standard errors clustered at the GP level. Continuous predictors are standardised (SD = 1); reference categories for binary variables are given in parentheses. Model 1: crude association. Model 2: adjusted for GP characteristics (sex, age, specialist status) and remuneration scheme. Model 3: additionally adjusted for patient list characteristics.

Table S2. Associations between GP sex and potential low-value MRI referral rates, Models 1–3.

Variable	Total MRI rate			Private MRI rate		
	**Model 1**	**Model 2**	**Model 3**	**Model 1**	**Model 2**	**Model 3**
GP male (female)	0.050*** (0.009)	0.044*** (0.009)	0.048*** (0.011)	0.063† (0.032)	0.029 (0.032)	0.109* (0.044)
GP age (per SD)		0.022*** (0.005)	0.002 (0.005)		0.059*** (0.017)	0.008 (0.016)
GP salaried (fee-for-service/capitation)		-0.104*** (0.014)	-0.050*** (0.015)		-0.740*** (0.079)	-0.095 (0.073)
GP specialist (non-specialist)		-0.077*** (0.010)	-0.056*** (0.009)		-0.076* (0.035)	-0.114*** (0.032)
Mean patient age (per SD)			0.019* (0.008)			0.088*** (0.027)
Share of female patients (per SD)			0.048*** (0.006)			0.079*** (0.020)
Share of patients with higher education (per SD)			-0.112*** (0.005)			-0.277*** (0.013)
Mean Charlson Comorbidity Index (per SD)			0.037*** (0.007)			-0.127*** (0.025)
Practice centrality (per SD)			0.097*** (0.006)			0.587*** (0.023)
R^2 (overall)	0.012	0.026	0.129	0.004	0.030	0.177
N (GP-years)	21 764	21 764	21 764	21 764	21 764	21 764

Note: β (standard error) shown. † p<0.1, * p<0.05, ** p<0.01, *** p<0.001. Outcomes are log-transformed crude referral rates to potentially low-value MRI examinations per 1000 listed patients. All models are pooled OLS with year fixed effects and standard errors clustered at the GP level. Continuous predictors are standardised (SD = 1); reference categories for binary variables are given in parentheses. Model 1: crude association. Model 2: adjusted for GP characteristics (sex, age, specialist status) and remuneration scheme (salaried vs. fee-for-service/capitation). Model 3: additionally adjusted for patient list characteristics.

Table S3. Associations between GP age and potential low-value MRI referral rates, Models 1–3.

Variable	Total MRI rate			Private MRI rate		
	**Model 1**	**Model 2**	**Model 3**	**Model 1**	**Model 2**	**Model 3**
GP age (per SD)	0.012** (0.004)	0.022*** (0.005)	0.002 (0.005)	0.063*** (0.015)	0.059*** (0.017)	0.008 (0.016)
GP male (female)		0.044*** (0.009)	0.048*** (0.011)		0.029 (0.032)	0.109* (0.044)
GP salaried (fee-for-service/capitation)		-0.104*** (0.014)	-0.050*** (0.015)		-0.740*** (0.079)	-0.095 (0.073)
GP specialist (non-specialist)		-0.077*** (0.010)	-0.056*** (0.009)		-0.076* (0.035)	-0.114*** (0.032)
Mean patient age (per SD)			0.019* (0.008)			0.088*** (0.027)
Share of female patients (per SD)			0.048*** (0.006)			0.079*** (0.020)
Share of patients with higher education (per SD)			-0.112*** (0.005)			-0.277*** (0.013)
Mean Charlson Comorbidity Index (per SD)			0.037*** (0.007)			-0.127*** (0.025)
Practice centrality (per SD)			0.097*** (0.006)			0.587*** (0.023)
R^2 (overall)	0.008	0.026	0.129	0.006	0.030	0.177
N (GP-years)	21 764	21 764	21 764	21 764	21 764	21 764

Note: β (standard error) shown. † p<0.1, * p<0.05, ** p<0.01, *** p<0.001. Outcomes are log-transformed crude referral rates to potentially low-value MRI examinations per 1000 listed patients. All models are pooled OLS with year fixed effects and standard errors clustered at the GP level. Continuous predictors are standardised (SD = 1); reference categories for binary variables are given in parentheses. Model 1: crude association. Model 2: adjusted for GP characteristics (sex, age, specialist status) and remuneration scheme (salaried vs. fee-for-service/capitation). Model 3: additionally adjusted for patient list characteristics.

Table S4. Associations between remuneration scheme and potential low-value MRI referral rates, Models 1–3.

Variable	Total MRI rate			Private MRI rate		
	Model 1	Model 2	Model 3	Model 1	Model 2	Model 3
GP salaried (fee-for-service/capitation)	-0.101*** (0.014)	-0.104*** (0.014)	-0.050*** (0.015)	-0.749*** (0.079)	-0.740*** (0.079)	-0.095 (0.073)
GP male (female)		0.044*** (0.009)	0.048*** (0.011)		0.029 (0.032)	0.109* (0.044)
GP age (per SD)		0.022*** (0.005)	0.002 (0.005)		0.059*** (0.017)	0.008 (0.016)
GP specialist (non-specialist)		-0.077*** (0.010)	-0.056*** (0.009)		-0.076* (0.035)	-0.114*** (0.032)
Mean patient age (per SD)			0.019* (0.008)			0.088*** (0.027)
Share of female patients (per SD)			0.048*** (0.006)			0.079*** (0.020)
Share of patients with higher education (per SD)			-0.112*** (0.005)			-0.277*** (0.013)
Mean Charlson Comorbidity Index (per SD)			0.037*** (0.007)			-0.127*** (0.025)
Practice centrality (per SD)			0.097*** (0.006)			0.587*** (0.023)
R^2 (overall)	0.012	0.026	0.129	0.028	0.030	0.177
N (GP-years)	21 764	21 764	21 764	21 764	21 764	21 764

Note: β (standard error) shown. † p<0.1, * p<0.05, ** p<0.01, *** p<0.001. Outcomes are log-transformed crude referral rates to potentially low-value MRI examinations per 1000 listed patients. All models are pooled OLS with year fixed effects and standard errors clustered at the GP level. Continuous predictors are standardised (SD = 1); reference categories for binary variables are given in parentheses. Model 1: crude association. Model 2: adjusted for GP characteristics (sex, age, specialist status) and remuneration scheme (salaried vs. fee-for-service/capitation). Model 3: additionally adjusted for patient list characteristics.

Table S5. Associations between hourly income from fees and potential low-value MRI referral rates, Models 1–3.

Variable	Total MRI rate			Private MRI rate		
	**Model 1**	**Model 2**	**Model 3**	**Model 1**	**Model 2**	**Model 3**
Hourly income from fees (per SD)	0.050*** (0.005)	0.047*** (0.005)	0.030*** (0.004)	0.158*** (0.014)	0.158*** (0.014)	0.066*** (0.013)
GP male (female)		0.032*** (0.009)	0.039** (0.012)		-0.013 (0.031)	0.088* (0.043)
GP age (per SD)		0.022*** (0.005)	0.002 (0.005)		0.061*** (0.017)	0.003 (0.016)
GP specialist in general practice (non-specialist)		-0.078*** (0.010)	-0.057*** (0.009)		-0.101** (0.034)	-0.128*** (0.032)
Mean patient age (per SD)			0.016* (0.008)			0.076** (0.026)
Share of female patients (per SD)			0.046*** (0.006)			0.073*** (0.020)
Share of patients with higher education (per SD)			-0.110*** (0.005)			-0.259*** (0.012)
Mean Charlson Comorbidity Index (per SD)			0.038*** (0.007)			-0.113*** (0.025)
Practice centrality (per SD)			0.086*** (0.006)			0.518*** (0.022)
R^2 (overall)	0.027	0.038	0.136	0.021	0.023	0.168
N (GP-years)	20 241	20 241	20 241	20 241	20 241	20 241

Note: β (standard error) shown. † p<0.1, * p<0.05, ** p<0.01, *** p<0.001. Outcomes are log-transformed crude referral rates to potentially low-value MRI examinations per 1000 listed patients. All models are pooled OLS with year fixed effects and standard errors clustered at the GP level. Continuous predictors are standardised (SD = 1); reference categories for binary variables are given in parentheses. Model 1: crude association. Model 2: adjusted for GP characteristics (sex, age, specialist status). Model 3: additionally adjusted for patient list characteristics. Hourly income from fees is estimated for fee-for-service/capitation GPs only; remuneration scheme (salaried vs. fee-for-service/capitation) is therefore not included in these models.

Table S6. Associations between consultation duration and potential low-value MRI referral rates, Models 1–3.

Variable	Total MRI rate			Private MRI rate		
	**Model 1**	**Model 2**	**Model 3**	**Model 1**	**Model 2**	**Model 3**
Short consultations <20 minutes (≥20 minutes)	0.089*** (0.009)	0.081*** (0.009)	0.040*** (0.009)	0.368*** (0.034)	0.287*** (0.035)	0.123*** (0.031)
GP male (female)		0.036*** (0.009)	0.043*** (0.011)		-0.001 (0.032)	0.095* (0.044)
GP age (per SD)		0.023*** (0.005)	0.004 (0.005)		0.061*** (0.017)	0.011 (0.016)
GP salaried (fee-for-service/capitation)		-0.072*** (0.014)	-0.037* (0.015)		-0.627*** (0.080)	-0.057 (0.074)
GP specialist in general practice (non-specialist)		-0.084*** (0.010)	-0.059*** (0.009)		-0.099** (0.035)	-0.124*** (0.032)
Mean patient age (per SD)			0.018* (0.007)			0.084** (0.027)
Share of female patients (per SD)			0.046*** (0.006)			0.075*** (0.020)
Share of patients with higher education (per SD)			-0.110*** (0.005)			-0.269*** (0.013)
Mean Charlson Comorbidity Index (per SD)			0.037*** (0.007)			-0.127*** (0.025)
Practice centrality (per SD)			0.093*** (0.006)			0.575*** (0.023)
R^2 (overall)	0.020	0.036	0.131	0.021	0.040	0.178
N (GP-years)	21 764	21 764	21 764	21 764	21 764	21 764

Note: β (standard error) shown. † $p<0.1$, * $p<0.05$, ** $p<0.01$, *** $p<0.001$. Outcomes are log-transformed crude referral rates to potentially low-value MRI examinations per 1000 listed patients. All models are pooled OLS with year fixed effects and standard errors clustered at the GP level. Continuous predictors are standardised (SD = 1); reference categories for binary variables are given in parentheses. Model 1: crude association. Model 2: adjusted for GP characteristics (sex, age, specialist status) and remuneration scheme (salaried vs. fee-for-service/capitation). Model 3: additionally adjusted for patient list characteristics.

Table S7. Associations between consultation frequency and potential low-value MRI referral rates, Models 1–3.

Variable	Total MRI rate			Private MRI rate		
	Model 1	Model 2	Model 3	Model 1	Model 2	Model 3
Consultation frequency (per SD)	0.078*** (0.004)	0.073*** (0.004)	0.048*** (0.004)	0.106*** (0.014)	0.078*** (0.014)	0.072*** (0.014)
GP male (female)		0.023** (0.009)	0.031** (0.011)		0.006 (0.032)	0.084† (0.044)
GP age (per SD)		0.027*** (0.005)	0.007 (0.005)		0.064*** (0.017)	0.014 (0.016)
GP salaried (fee-for-service/capitation)		-0.058*** (0.014)	-0.018 (0.015)		-0.691*** (0.079)	-0.047 (0.074)
GP specialist in general practice (non-specialist)		-0.070*** (0.010)	-0.054*** (0.009)		-0.068† (0.035)	-0.111*** (0.032)
Mean patient age (per SD)			0.021** (0.007)			0.091*** (0.026)
Share of female patients (per SD)			0.042*** (0.006)			0.071*** (0.020)
Share of patients with higher education (per SD)			-0.099*** (0.005)			-0.257*** (0.013)
Mean Charlson Comorbidity Index (per SD)			0.032*** (0.007)			-0.134*** (0.025)
Practice centrality (per SD)			0.092*** (0.006)			0.580*** (0.023)
R^2 (overall)	0.053	0.064	0.144	0.010	0.034	0.179
N (GP-years)	21 764	21 764	21 764	21 764	21 764	21 764

Note: β (standard error) shown. † p<0.1, * p<0.05, ** p<0.01, *** p<0.001. Outcomes are log-transformed crude referral rates to potentially low-value MRI examinations per 1000 listed patients. All models are pooled OLS with year fixed effects and standard errors clustered at the GP level. Continuous predictors are standardised (SD = 1); reference categories for binary variables are given in parentheses. Model 1: crude association. Model 2: adjusted for GP characteristics (sex, age, specialist status) and remuneration scheme (salaried vs. fee-for-service/capitation). Model 3: additionally adjusted for patient list characteristics.

Table S8. Associations between patient list size and potential low-value MRI referral rates, Models 1–3.

	Total MRI rate			Private MRI rate		
**Variable**	**Model 1**	**Model 2**	**Model 3**	**Model 1**	**Model 2**	**Model 3**
Patient list size (per SD)	0.030*** (0.005)	0.024*** (0.005)	0.032*** (0.005)	0.266*** (0.015)	0.240*** (0.016)	0.050*** (0.014)
GP male (female)		0.038*** (0.009)	0.037** (0.011)		-0.038 (0.032)	0.092* (0.044)
GP age (per SD)		0.018*** (0.005)	-0.002 (0.005)		0.020 (0.017)	0.001 (0.016)
GP salaried (fee-for-service/capitation)		-0.080*** (0.015)	-0.036* (0.015)		-0.502*** (0.079)	-0.074 (0.074)
GP specialist in general practice (non-specialist)		-0.082*** (0.010)	-0.059*** (0.009)		-0.122*** (0.035)	-0.118*** (0.032)
Mean patient age (per SD)			0.017* (0.007)			0.085** (0.027)
Share of female patients (per SD)			0.048*** (0.006)			0.080*** (0.020)
Share of patients with higher education (per SD)			-0.114*** (0.005)			-0.279*** (0.013)
Mean Charlson Comorbidity Index (per SD)			0.040*** (0.007)			-0.121*** (0.026)
Practice centrality (per SD)			0.083*** (0.006)			0.566*** (0.024)
R^2 (overall)	0.014	0.030	0.135	0.051	0.063	0.178
N (GP-years)	21 764	21 764	21 764	21 764	21 764	21 764

Note: β (standard error) shown. † p<0.1, * p<0.05, ** p<0.01, *** p<0.001. Outcomes are log-transformed crude referral rates to potentially low-value MRI examinations per 1000 listed patients. All models are pooled OLS with year fixed effects and standard errors clustered at the GP level. Continuous predictors are standardised (SD = 1); reference categories for binary variables are given in parentheses. Model 1: crude association. Model 2: adjusted for GP characteristics (sex, age, specialist status) and remuneration scheme (salaried vs. fee-for-service/capitation). Model 3: additionally adjusted for patient list characteristics.

Table S9. Associations between patient list morbidity and potential low-value MRI referral rates, Models 1–3.

Variable	Total MRI rate			Private MRI rate		
	**Model 1**	**Model 2**	**Model 3**	**Model 1**	**Model 2**	**Model 3**
Mean Charlson Comorbidity Index (per SD)	0.065*** (0.004)	0.063*** (0.005)	0.037*** (0.007)	-0.124*** (0.017)	-0.136*** (0.018)	-0.127*** (0.025)
GP male (female)		0.010 (0.009)	0.048*** (0.011)		0.104** (0.033)	0.109* (0.044)
GP age (per SD)		0.014** (0.005)	0.002 (0.005)		0.076*** (0.017)	0.008 (0.016)
GP salaried (fee-for-service/capitation)		-0.126*** (0.014)	-0.050*** (0.015)		-0.693*** (0.077)	-0.095 (0.073)
GP specialist in general practice (non-specialist)		-0.067*** (0.010)	-0.056*** (0.009)		-0.097** (0.035)	-0.114*** (0.032)
Mean patient age (per SD)			0.019* (0.008)			0.088*** (0.027)
Share of female patients (per SD)			0.048*** (0.006)			0.079*** (0.020)
Share of patients with higher education (per SD)			-0.112*** (0.005)			-0.277*** (0.013)
Practice centrality (per SD)			0.097*** (0.006)			0.587*** (0.023)
R^2 (overall)	0.041	0.056	0.129	0.014	0.041	0.177
N (GP-years)	21 764	21 764	21 764	21 764	21 764	21 764

Note: β (standard error) shown. † $p<0.1$, * $p<0.05$, ** $p<0.01$, *** $p<0.001$. Outcomes are log-transformed crude referral rates to potentially low-value MRI examinations per 1000 listed patients. All models are pooled OLS with year fixed effects and standard errors clustered at the GP level. Continuous predictors are standardised (SD = 1); reference categories for binary variables are given in parentheses. Model 1: crude association. Model 2: adjusted for GP characteristics (sex, age, specialist status) and remuneration scheme (salaried vs. fee-for-service/capitation). Model 3: additionally adjusted for patient list characteristics.
